# Multilocus sequence typing method for identification and genotypic classification of pathogenic *Leptospira *species

**DOI:** 10.1186/1476-0711-5-28

**Published:** 2006-11-23

**Authors:** Niyaz Ahmed, S Manjulata Devi, M de los Á Valverde, P Vijayachari, Robert S Machang'u, William A Ellis, Rudy A Hartskeerl

**Affiliations:** 1 Pathogen Evolution Group, Centre for DNA Fingerprinting and Diagnostics (CDFD), Hyderabad 500076, India; 2 ISOGEM working group on Spirochetes, The International Society for Genomic and Evolutionary Microbiology (ISOGEM), Sassari, Italy; 3 National Reference Center Leptospirosis. INCIENSA (Costarrican Institute for Research in Nutrition and Health), Costa Rica; 4 Regional Medical Research Centre (RMRC), Port Blair, India; 5 Department of Veterinary Microbiology and Parasitology, Sokoine University of Agriculture, P. O. Box 3019, Morogoro, Tanzania; 6 Veterinary Sciences Division (VSD), The Queen's University of Belfast, Stoney Road, Stormont, Belfast, Northern Ireland, BT4 3SD, UK; 7 WHO/FAO/OIE and National Collaborating Centre for Reference and Research on Leptospirosis, KIT Biomedical Research, KIT (Koninklijk Instituut voor de Tropen/Royal Tropical Institute) Meibergdreef 39, 1105 AZ Amsterdam, The Netherlands

## Abstract

**Background:**

*Leptospira *are the parasitic bacterial organisms associated with a broad range of mammalian hosts and are responsible for severe cases of human Leptospirosis. The epidemiology of leptospirosis is complex and dynamic. Multiple serovars have been identified, each adapted to one or more animal hosts. Adaptation is a dynamic process that changes the spatial and temporal distribution of serovars and clinical manifestations in different hosts. Serotyping based on repertoire of surface antigens is an ambiguous and artificial system of classification of leptospiral agents. Molecular typing methods for the identification of pathogenic leptospires up to individual genome species level have been highly sought after since the decipherment of whole genome sequences. Only a few resources exist for microbial genotypic data based on individual techniques such as Multiple Locus Sequence Typing (MLST), but unfortunately no such databases are existent for leptospires.

**Results:**

We for the first time report development of a robust MLST method for genotyping of *Leptospira*. Genotyping based on DNA sequence identity of 4 housekeeping genes and 2 candidate genes was analyzed in a set of 120 strains including 41 reference strains representing different geographical areas and from different sources. Of the six selected genes, *adk*, *icd*A and *sec*Y were significantly more variable whereas the LipL32 and LipL41 coding genes and the *rrs*2 gene were moderately variable. The phylogenetic tree clustered the isolates according to the genome-based species.

**Conclusion:**

The main advantages of MLST over other typing methods for leptospires include reproducibility, robustness, consistency and portability. The genetic relatedness of the leptospires can be better studied by the MLST approach and can be used for molecular epidemiological and evolutionary studies and population genetics.

## Background

Leptospirosis is a zoonotic and an emerging infectious disease caused by the pathogenic *Leptospira *species and is identified in the recent years as a global public health problem because of its increased mortality and morbidity in different countries. Leptospirosis is frequently misdiagnosed as a result of its protean and non-specific presentation resembling many other febrile diseases, notably viral haemorrhagic fevers such as dengue [1]. There is, for certain, an underestimation of the leptospirosis problem due to lack of awareness and under-recognition through a lack of proper use of diagnostic tools.

The common mode of transmission of the infection in humans is either by direct or indirect contact with the urine of infected animals and may lead to potential lethal disease. A unique feature of this organism is to parasitize in a wide variety of wild and domestic animals [2]. Traditionally, two species have been identified, i.e. *Leptospira interrogans *and *L. biflexa *for pathogenic and non-pathogenic leptospires, respectively. The serovar is the basic identifier, characterized on the basis of serological criteria. To date nearly 300 serovars have been identified under the species *L. interrogans *alone that have been distributed among 25 different serogroups of antigenically similar serovars [3].

Previously a classification system based on DNA-DNA hybridization studies has been introduced, which now comprises 17 *Leptospira *species [4-7]. Among these, 7 species: *L. interrogans*, *L. borgpetersenii*, *L. santarosai*, *L. noguchii*, *L. weilli*, *L. kirschneri and L. alexanderi *are considered as the main agents of leptospirosis [5,6]. The enormous inventory of serovars, based mainly on an ever-changing surface antigen repertoire, throws an artificial and unreliable scenario of strain diversity. It is therefore difficult to track strains whose molecular identity keeps changing according to the host and the environmental niches they inhabit and cross through.

Other than the serological methods, molecular tools that have been employed so far for sub-classification and cataloguing of leptospiral agents include restriction endonuclease assay (REA) [8,9], pulsed field gel electrophoresis (PFGE) [10,11], restriction fragment length polymorphism (RFLP) [12], arbitrarily primed PCR [13], Variable Number of Tandem Repeats (VNTR) analysis [14] and fluorescent amplified fragment length polymorphism (FAFLP) [15]. All these techniques however, suffer from certain disadvantages that include requirement of large quantity of pure and high quality DNA, low discriminatory power, low reproducibility, ambiguous interpretation of data and problems associated with transfer of data between different laboratories [14].

MLST is a simple PCR based technique, which makes use of automated DNA sequencers to assign and characterize the alleles present in different target genes. The method allows one to generate sequence data in a low to high-throughput scale, which is unambiguous and suitable for epidemiological and population studies. The selected loci are generally the housekeeping genes, which evolve very slowly over an evolutionary time-scale [16] and hence qualify as highly robust markers of ancient and modern ancestry. The sequencing of multiple loci provides a balance between technical feasibility and resolution. MLST has been applied to the study of many other bacterial species like *Neisseria meningitides *[17], *Streptococcus pneumoniae *[18], *Yersinia *species [19], *Campylobacter jejuni *[20] and *Helicobacter pylori *[21].

Our present study is the first attempt to use the MLST, which currently differentiates the species and examines the intra and interspecies relationships of *Leptospira*. This method in future could be developed as a highly sophisticated genotyping system based on integrated genome analysis approaches to correctly identify and track leptospiral strains and is expected to greatly facilitate epidemiology of leptospirosis apart from deciphering the origins and evolution of leptospires in a global sense.

## Methods

### Bacterial strains

Bacterial strains (Table 1) were cultured by the WHO reference laboratory at the KIT Biomedical Research Centre at The Royal Tropical Institute, Amsterdam, The Netherlands (all isolates and reference strains labelled RK3) and at the Veterinary Sciences Division (VSD), The Queen's University of Belfast, United Kingdom (reference strains labelled RB3) and the WHO reference centre at Port Blair India (labelled isol 15). A total of 120 strains consisting of 79 isolates and 41 reference strains from different sources and geographical regions were analyzed by MLST. The 41 reference strains included in the study belonged to six *Leptospira *species (*L. interrogans*; *L. kirschneri*;* L. noguchii*; *L. borgpetersenii*; *L. santarosai *and *L. alexanderi*).

**Table 1:** Details of leptospiral strains and isolates used for MLST based

**Labels**	**Genome Species**	**Serogroup**	**Serovar**	**Strain**	**Geographical area**	**Source**
INT 01	*L. interrogans*	Canicola	Sumneri	Sumner	Malaysia	RB3
INT 02	*L. interrogans*	Canicola	Portlandvere	MY 1039	Jamaica	RB3
INT 03	*L. interrogans*	Pomona	Pomona	Pomona	Australia	RB3
INT 04	*L. interrogans*	Pomona	Proechimys	1161 U	Panama	RB3
INT 05	*L. interrogans*	Pomona	Kenniwicki	LT 1026	USA	RB3
INT 06	*L. interrogans*	Grippotyphosa	Grippotyphosa	Moskva V	Unknown	RB4
INT 07	*L. interrogans*	Grippotyphosa	Muelleri	RM 2	Malaysia	RB3
INT 08	*L. interrogans*	Sejroe	Roumanica	LM 294	Roumania	RB3
INT 09	*L. interrogans*	Sejroe	Saxkoebing	Mus 24	Denmark	RB3
INT 10	*L. interrogans*	Sejroe	Hardjoprajitno	Hardjoprajitno	Indonesia	RB3
INT 11	*L. interrogans*	Icterohaemorrhagiae	Lai	Lai	China	RB3
INT 12	*L. interrogans*	Icterohaemorrhagiae	Copenhageni	M 20	Denmark	RB3
INT 13	*L. interrogans*	Grippotyphosa	Valbuzzi	Valbuzzi	Australia	RB3
INT 14	*L. interrogans*	Pyrogenes	Manilae	LT 398	Phillipins	RB3
INT 15	*L. interrogans*	Australis	Australis	Ballico	Ballico	RK3
INT 16	*L. interrogans*	Icterohaemorrhagiae	Icterohaemorrhagiae	RGA	Germany	RK3
INT 17	*L. interrogans*	Grippotyphosa	Ratnapura	Field Isolate 1	South Andaman	Isol 15
INT 18	*L. interrogans*	Icterohaemorrhagiae	Copenhageni	Field Isolate 2	South Andaman	Isol 15
INT 19	*L. interrogans*	Grippotyphosa	Ratnapura	Field Isolate 3	South Andaman	Isol 15
INT 20	*L. interrogans*	Grippotyphosa	Ratnapura	Field Isolate 4	South Andaman	Isol 15
INT 21	*L. interrogans*	Grippotyphosa	Valbuzzi	Field Isolate 5	South Andaman	Isol 15
INT 22	*L. interrogans*	Icterohaemorrhagiae	Copenhageni	Field Isolate 6	South Andaman	Isol 15
INT 23	*L. interrogans*	Grippotyphosa	Valbuzzi	Field Isolate 7	North Andaman	Isol 15
INT 24	*L. interrogans*	Grippotyphosa	Valbuzzi	Field Isolate 8	North Andaman	Isol 15
INT 25	*L. interrogans*	Grippotyphosa	Ratnapura	Field Isolate 9	South Andaman	Isol 15
INT 26	*L. interrogans*	Grippotyphosa	Ratnapura	Field Isolate 10	South Andaman	Isol 15
INT 27	*L. interrogans*	Grippotyphosa	Ratnapura	Field Isolate 11	South Andaman	Isol 15
INT 28	*L. interrogans*	Grippotyphosa	Unknown	Field Isolate 12	South Andaman	Isol 15
INT 29	*L. interrogans*	Grippotyphosa	Unknown	Field Isolate 13	South Andaman	Isol 15
INT 30	*L. interrogans*	Sejroe	Sejroe	Field Isolate 14	South Andaman	Isol 15
INT 31	*L. interrogans*	Pomona	Unknown	Field Isolate 15	South Andaman	Isol 15
INT 32	*L. interrogans*	Grippotyphosa	Ratnapura	Field Isolate 16	South Andaman	Isol 15
INT 33	*L. interrogans*	Australis	Ramisi	Field Isolate 17	South Andaman	Isol 15
INT 34	*L. interrogans*	Grippotyphosa	Unknown	Field Isolate 18	South Andaman	Isol 15
INT 35	*L. interrogans*	Grippotyphosa	Valbuzzi	Field Isolate 19	South Andaman	Isol 15
INT 36	*L. interrogans*	Grippotyphosa	Valbuzzi	Field Isolate 20	South Andaman	Isol 15
INT 37	*L. interrogans*	Hebdomadis	Goiano	Bovino 131	Brazil	RB3
INT 38	*L. interrogans*	Canicola*	Canicola*	M12/90	Brazil	Isol
INT 39	*L. interrogans*	Icterohaemorrhagiae*	Copenhageni*	M9/99	Brazil	Isol
INT 40	*L. interrogans*	Australis*	Rushan*	L01	Brazil	Isol
INT 41	*L. interrogans*	Canicola*	Canicola*	L02	Brazil	Isol
INT 42	*L. interrogans*	Canicola*	Canicola*	L03	Brazil	Isol
INT 43	*L. interrogans*	Canicola*	Canicola*	L09	Brazil	Isol
INT 44	*L. interrogans*	Icterohaemorrhagiae*	Copenhageni*	L10	Brazil	Isol
INT 45	*L. interrogans*	Canicola*	Canicola*	L14	Brazil	Isol
INT 46	*L. interrogans*	Lyme*	Lyme*	K30B	UK	Isol
INT 47	*L. interrogans*	Australis*	Australis*	K9H	UK	Isol
INT 48	*L. interrogans*	Icterohaemorrhagiae*	Copenhageni*	Isolate 9	Costa Rica	Isol
INT 49	*L. interrogans*	Unknown*	Unknown*	Isolate 10	Costa Rica	Isol
INT 50	*L. interrogans*	Australis*	Lora*	1992	Tanzania	Isol
INT 51	*L. interrogans*	Australis*	Lora*	2324	Tanzania	Isol
INT 52	*L. interrogans*	Australis*	Lora*	2364	Tanzania	Isol
INT 53	*L. interrogans*	Australis*	Lora*	2366	Tanzania	Isol
INT 54	*L. interrogans*	Ballum*	Kenya*	4885	Tanzania	Isol
INT 55	*L. interrogans*	Ballum*	Kenya*	4883	Tanzania	Isol
KIR 01	*L. kirschneri*	Canicola	Kuwait	136/2/2	Kuwait	RB3
KIR 02	*L. kirschneri*	Canicola	Schueffneri	Vleermuis 90 C	Indonesia	RB3
KIR 03	*L. kirschneri*	Pomona	Mozdok	5621	Soviet Union (Russia)	RB3
KIR 04	*L. kirschneri*	Grippotyphosa	Vanderhoedeni	Kipod 179	Israel	RB3
KIR 05	*L. kirschneri*	Pomona	Tsaratsovo	B 81/7	Bulgaria	RB3
KIR 06	*L. kirschneri*	Grippotyphosa	Grippotyphosa	Moskva V	Russia	RK3
KIR 07	*L. kirschneri*	Grippotyphosa	Ratnapura	Wumalasena	Sri Lanka	RK3
KIR 08	*L. kirschneri*	Icterohaemorrhagiae*	Sokoine*	745	Tanzania	Isol
KIR 09	*L. kirschneri*	Icterohaemorrhagiae*	Sokoine*	771	Tanzania	Isol
KIR 10	*L. kirschneri*	Icterohaemorrhagiae*	Mwogolo*	826	Tanzania	Isol
KIR 11	*L. kirschneri*	Icterohaemorrhagiae*	Mwogolo*	845	Tanzania	Isol
KIR 12	*L. kirschneri*	Canicola*	Qunjian*	2980	Tanzania	Isol
KIR 13	*L. kirschneri*	Icterohaemorrhagiae*	Sokoine*	4602	Tanzania	Isol
KIR 14	*L. kirschneri*	Sejroe*	Ricardi/Saxkoebing*	1499	UK	Isol
KIR 15	*L. kirschneri*	Sejroe*	Ricardi/Saxkoebing*	1501	UK	Isol
KIR 16	*L. kirschneri*	Ballum*	Kenya	Njenga	Kenya	RK3
NOG 01	*L. noguchii*	Pyrogenes	Myocastoris	LSU 1551	USA	RB3
NOG 02	*L. noguchii*	Louisiana	Louisiana	LSU 1945	USA	RK3
NOG 03	*L. noguchii*	Panama	Panama	CZ214k	Panama	RK3
NOG 04	*L. noguchii*	Pyrogenes*	Guaratuba *	Isolate 4	Costa Rica	Isol
SAN 01	*L. santarosai*	Mini	Georgia	LT 117	USA	RB3
SAN 02	*L. santarosai*	Sejroe	Recreo	380	Nicaragua	RB3
SAN 03	*L. santarosai*	Pyrogenes	Guaratuba	An 7705	Brazil	RB3
SAN 04	*L. santarosai*	Pyrogenes	Varela	1019	Nicaragua	RB3
SAN 05	*L. santarosai*	Grippotyphosa	Canalzonae	CZ188	Panama	RK3
SAN 06	*L. santarosai*	Bataviae*	Brasiliensis*	An 776	Brazil	Isol
SAN 07	*L. santarosai*	Sejroe*	Guaricura*	Bov.G	Brazil	Isol
SAN 08	*L. santarosai*	Sejroe*	Guaricura*	M4/98	Brazil	Isol
SAN 09	*L. santarosai*	Grippotyphosa*	Bananal*	2ACAP	Brazil	Isol
SAN 10	*L. santarosai*	Grippotyphosa*	Bananal*	16CAP	Brazil	Isol
SAN 11	*L. santarosai*	Pyrogenes*	Alexi/Guaratuba/Princestown*	Isolate 1	Costa Rica	Isol
SAN 12	*L. santarosai*	Sarmin*	Weaveri/Rio*	Isolate 2	Costa Rica	Isol
SAN 13	*L. santarosai*	Tarassovi*	Rama*	Isolate 3	Costa Rica	Isol
SAN 14	*L. santarosai*	Tarassovi*	Rama*	Isolate 5	Costa Rica	Isol
SAN 15	*L. santarosai*	Bataviae*	Claytoni*	Isolate 6	Costa Rica	Isol
SAN 16	*L. santarosai*	Shermani*	Shermani/Babudieri/Aguaruna*	Isolate 8	Costa Rica	Isol
SAN 17	*L. santarosai*	unknown*	(putative new serovar)#	Isolate 7	Costa Rica	Isol
SAN 18	*L. santarosai*	Icterohaemorrhagiae*	Copenhageni*	K13A	UK	Isol
ALE 01	*L. alexanderi*	Manhao	Manhao	L60	China	RK3
BOR 01	*L. borgpetersenii*	Sejroe	Istarica	Bratislava	Slovakia	RB3
BOR 02	*L. borgpetersenii*	Sejroe	Sejroe	M 84	Denmark	RB3
BOR 03	*L. borgpetersenii*	Javanica	Dehong	De 10	China	RB3
BOR 04	*L. borgpetersenii*	Javanica	Javanica	Veltrat Batavia	Indonesia	RB3
BOR 05	*L. borgpetersenii*	Javanica	Zhenkang	L 82	China	RB3
BOR 06	*L. borgpetersenii*	Javanica	Poi	Poi	Italy	RK3
BOR 07	*L. borgpetersenii*	Mini	Mini	Sari	Italy	RK3
BOR 08	*L. borgpetersenii*	Ballum*	Kenya*	153	Tanzania	Isol
BOR 09	*L. borgpetersenii*	Ballum *	Kenya*	159	Tanzania	Isol
BOR 10	*L. borgpetersenii*	Ballum *	Kenya*	723	Tanzania	Isol
BOR 11	*L. borgpetersenii*	Ballum *	Kenya*	766	Tanzania	Isol
BOR 12	*L. borgpetersenii*	Ballum *	Kenya*	1605	Tanzania	Isol
BOR 13	*L. borgpetersenii*	Ballum *	Kenya*	1610	Tanzania	Isol
BOR 14	*L. borgpetersenii*	Ballum *	Kenya*	2062	Tanzania	Isol
BOR 15	*L. borgpetersenii*	Ballum *	Kenya*	2348	Tanzania	Isol
BOR 16	*L. borgpetersenii*	Ballum *	Kenya*	2447	Tanzania	Isol
BOR 17	*L. borgpetersenii*	Ballum *	Kenya*	4880	Tanzania	Isol
BOR 18	*L. borgpetersenii*	Ballum *	Kenya*	4787	Tanzania	Isol
BOR 19	*L. borgpetersenii*	Hebdomadis*	Kremastos/Hebdomadis*	873	Ireland	Isol
BOR 20	*L. borgpetersenii*	Hebdomadis*	Kremastos/Hebdomadis*	871	Ireland	Isol
BOR 21	*L. borgpetersenii*	Sejroe*	Saxkoebing*	1498	Ireland	Isol
BOR 22	*L. borgpetersenii*	Sejroe*	Ricardi/Saxkoebing*	1522	UK	Isol
BOR 23	*L. borgpetersenii*	Sejroe*	Ricardi/Saxkoebing*	1525	UK	Isol
BOR 24	*L. borgpetersenii*	Pomona*	Kunming*	RIM 139	Portugal	Isol
BOR 25	*L. borgpetersenii*	Pomona*	Kunming*	RIM 201	Portugal	Isol
BOR 26	*L. borgpetersenii*	Sejroe*	Ricardi/Saxkoebing*	RIM 156	Portugal	Isol

* – Unpublished presumptive classification, # – Unpublished putative new serovar, Isol – Isolates, RB – reference strains from Belfast lab, RK – reference strains from KIT. The numbers 3, 4 and 15 refer to the references describing strains or isolates.

### Selection and validation of target genes for MLST

The candidate loci sequences were obtained from the strains *L. interrogans *Fiocruz L1-130 and *L. interrogans *Lai 56601 strains from the Leptolist server. Six genes, namely *adk *(Adenylate Kinase), *icd*A (Isocitrate dehydrogenase), LipL32 (outer membrane lipoprotein LipL32), *rrs*2 (16S rRNA), *sec*Y (pre-protein translocase SecY protein), and LipL41 (outer membrane Lipoprotein LipL41) (Table 2) were selected for MLST analysis. Many sequences of the *rrs2*, LipL32 and LipL41 are available in the GenBank [2]. PCR primers were designed for these genes based on GenBank records in the conserved regions flanking the variable internal fragments of the target regions. PCR primers for *adk*, *icd*A and *sec*Y were based on gene sequences of strains Fiocruz L1-130 and Lai 56601 [22,23] (Table 2). The Primer 3 software [24] was used to design the PCR primers for the amplification of the candidate loci. The PCR amplifications of the different MLST target genes were performed using 1.5 mM MgCl_2_, 200 μM of dNTP's (MBI Fermentas), 25–50 ng template DNA using Gene Amp 9700 (Applied Biosystems, Foster City, USA) PCR system.

**Table 2:** Details of gene loci and the corresponding primer sequences used for MLST analysis

**Gene**	**Locus**	**Gene size (bp) **	**Co-ordinates**	**PCR product size (bp)**	**Size of polymorphic sequence (bp)**	**Function**	**Primer sequences**
adk	LIC12852	564	3458298–3458861	531	430	Adenylate Kinase	F-GGGCTGGAAAAGGTACACAA
							R-ACGCAAGCTCCTTTTGAATC
icdA	LIC13244	1197	3979829–3981025	674	557	Isocitarate Dehydrogenase	F-GGGACGAGATGACCAGGAT
							R-TTTTTTGAGATCCGCAGCTTT
LipL41	LIC12966	1068	3603575–3604642	520	518	Outermenbrane Lipoprotein LipL41	F-TAGGAAATTGCGCAGCTACA
							R-GCATCGAGAGGAATTAACATCA
rrs2	LIC11508	1512	1862433–1863944	541	452	16S ribosomal RNA	F-CATGCAAGTCAAGCGGAGTA
							R-AGTTGAGCCCGCAGTTTTC
secY	LIC12853	1383	3458869–3460251	549	549	Translocase pre-protein secY	F-ATGCCGATCATTTTTGCTTC
							R-CCGTCCCTTAATTTTAGACTTCTTC
LipL32	LIC11352	819	1666299–1667117	474	474	Outermenbrane Lipoprotein LipL32	F-ATCTCCGTTGCACTCTTTGC
							R-ACCATCATCATCATCGTCCA

Amplification parameters included an initial denaturation at 95°C for 5 min followed by 35 cycles of amplification comprising of denaturation (94°C for 30 sec), annealing (58°C for 30 sec) and primer extension (72°C for 1 min) steps and a final extension of 7 min at 72°C. All the amplified fragments were checked on 1.5% or 2% agarose gel with ethidium bromide staining and the amplicons were sequenced in both the directions using Big Dye Terminator cycle sequencing Kit (Applied Biosystems, Foster City, USA) on ABI 3100 DNA sequencers (Applied Biosystems, Foster City, USA).

### MLST data analysis

The electropherograms were viewed by using Chromas Lite version 2.01 (Technelysium Pty Ltd, Australia) and the resulting DNA sequences corresponding to both the forward and reverse reads were aligned using the Seqscape software (Applied Biosystems, Foster City, USA). Low quality nucleotide sequences were trimmed from the ends while comparing with the reference sequence of the Fiocruz strain and all the processed sequences were subsequently aligned by Clustal X [25]. The Sequence Type Analysis and Recombinational Test (START) programme [26] was used to determine Guanine-Cytosine content, number of polymorphic sites and the ratio of non-synonymous to synonymous nucleotide substitutions (d_N_/d_S_). The phylogenetic analysis was performed using concatenated (2980bp) sequences in the order *adk*, *icd*A, LipL32, LipL41, *rrs*2 and *sec*Y for each strain using MEGA 3.1 [27] and the consensus tree was drawn based on 1000 bootstrap replicates with Kimura 2 parameter.

## Results

### Diversity among the candidate loci analyzed

The 5' parts of *rrs*2, LipL32, LipL41 and the 3' part of *sec*Y were considered for the analysis based on abundance of nucleotide substitution positions found in these regions. The sizes of the fragments analyzed for the selected housekeeping genes ranged between 430bp (*adk*) and 557bp (*icd*A). The positions of these MLST loci were scattered throughout the chromosome I of *L. interrogans *Fiocruz L1-130 (Table 2). Clustal X programme was used to align all the individual sequences separately and we observed that there were no large insertions and deletions in the selected region. According to our analysis the *rrs*2 gene was found to be highly conserved among all the isolates with the percentage of variable sites being 4.42. Other genes namely LipL32, LipL41, *icd*A, *adk *and *sec*Y, however, were significantly diverse with the percentages of variable sites being 11.3, 21.04, 22.8, 27.2 and 28.7 respectively. The locus with highest diversity was *icd*A with 51 different alleles found among the set of 120 different isolates studied. The ratio of non-synonymous (d_N_) to synonymous substitution (d_S_) was much less than 1.0 indicating that these genes are not under positive selection pressure (the selection is against the amino acid change), whereas the *rrs*2 gene showed d_N_/d_S _ratio as 1.369 suggesting a high flexibility for amino acid changes. The percentage of G + C content in these loci ranged from 39.16 (*sec*Y) to 51.92 (*rrs*2) (Table 3). The synonymous substitution which, plays a role in the divergence of strains was more frequent in *icd*A and *sec*Y with 126 different synonymous sites. When compared to synonymous substitutions, non-synonymous substitutions were more frequent in all the genes tested, but highest numbers of 429 and 423 were observed in case of *icd*A and *sec*Y respectively (Table 3).

**Table 3:** Allelic diversity parameters observed for the six target genes used for MLST analysis of leptospires

**Gene **	**G+C%**	**No. of alleles**	**Polymorphic sites**	**Synonymous sites**	**Non-synonymous sites**	**% of variable nucleotide sites**	**d**_**N**_**/d**_**S**_**ratio**
*adk*	41.55	40	117	100	329	27.2	0.039
*icd*1	40.9	51	127	126	429	22.8	0.017
LipL32	46.46	36	54	112	362	11.3	0.091
LipL41	42.88	52	109	123	393	21.04	0.055
*rrs*2	51.92	29	20	112	338	4.42	1.369
*sec*Y	39.16	49	158	126	423	28.7	0.019

### Clustering analysis of Leptospires based on MLST

**Figure 1:**
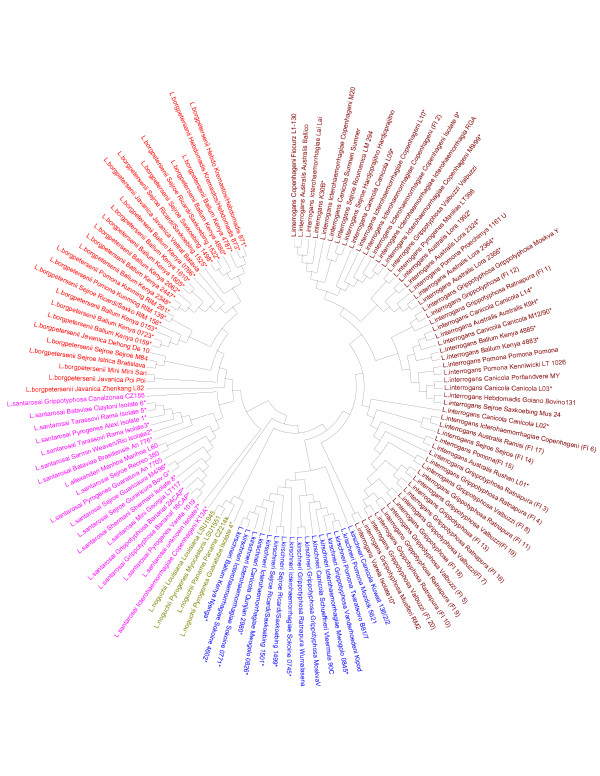
Genetic relatedness among *Leptospira *isolates based on the concatenated sequences of the six housekeeping and candidate gene loci analyzed (see table 1 for detailed information on isolates/strains). * Unpublished presumptive serological classification.

The neighbor-joining tree was constructed for representative isolates based on a 'super locus' of 2980bp comprising concatenated sequence of all the six loci. For this, the genes were fused in the order – *ad*k, *icd*A, LipL32, LipL41, *rrs*2 and *sec*Y. The phylogenetic tree generated five different clusters where *L. interrogans *(56 samples), *L. noguchii *(4 samples), *L. kirschneri *(16 samples), *L. santarosai *(18 samples), *L. alexanderi *(1 sample), *L. borgpetersenii *(26 samples) separated according to their genome species (Figure 1).

MLST analysis also clearly identified each of the field isolates up to the species level and in general, classification based on these observations corroborated with previous taxonomic status of these isolates determined either by serological criteria or by genomic methods such as FAFLP (data not shown). There are two isolates for which serological classification seemed to be in contrast to MLST identification, i.e. INT 46, *L. interrogans *serovar Lyme and SAN 18, *L. santarosai *serovar Copenhageni. It should be noted that in these cases serovar designation is based on preliminary serological analysis, which may be incorrect. *L. alexanderi *was found to be genomically highly similar to *L. santarosai *and clustered accordingly. This could therefore be a subspecies of *L. santarosai*.

*L. interrogans *isolate SAN 17 from Costa Rica, indicated as putative new serovar (Table 1) along with another *L. interrogans *member belonging to serovar Muelleri of the serogroup Grippotyphosa, formed an isolated branch under the *L. interrogans *cluster arguing for a separate taxonomic status, possibly another subspecies of *L. interrogans*.

## Discussion

The present study was a first attempt in the development of MLST for *Leptospira *species; the main objective being the selection of the housekeeping and candidate genes that are species specific, stable and evolve slowly. The availability of the complete sequence of *L. interrogans *Lai 56601 and Fiocruz L1-130 helped us in selecting the candidate loci. Genetically diverse group of strains was used for the study to evaluate the sequence diversity among the tested housekeeping genes. The six genes selected and studied here appear to be distinctly resolving to reveal a wide variety of genotypes among the isolates analyzed. This indicates a significant heterogeneity and sequence variation at each locus (Table 3).

The six loci selected were found to be suitable for MLST typing as they can be amplified and sequenced in all the isolates irrespective of species as these loci are unlinked on the *L. interrogans *chromosome I and exhibit a modest degree of sequence diversity and resolution. A total of 585 polymorphic sites were observed in the 'super locus' of 2980bp. Non-synonymous sites were more abundant as compared to synonymous sites (Table 3) indicating that the amino acid sequence variability possibly represents acclimatization to the specific host and environmental restrictions [2].

Several molecular tools that have been so far described for the characterization of *Leptospira *are associated with several drawbacks. Methods like PFGE, RFLP, and REA need large quantity of purified DNA, present tedious methodology, have low discriminatory levels, are hard to interpret the data, suffer from lack of reproducibility, require specialized equipment such as counter clamped homogenous electric field electrophoresis systems and give poor data transfer. The VNTR or MLVA technique described by Majed *et al *[14] and Slack *et al *[28] are more specific to *L. interrogans*. MLST overcomes all these disadvantages as this technique is simple, and easy to standardize on an automated DNA sequencer that is more widely available in most of the laboratories and above all the sequence data generated are unambiguous, specific and explicit. The main advantage of MLST is the transfer of data that can be shared and compared between different laboratories easily through the Internet. To date, a large number of organisms have been typed by MLST, which proved to be a highly discriminatory technique [29]. MLST analysis on *Leptospira *strains showed that the similar serovars and the serogroups of different species are not clustered together (Figure 1). This method is more suitable in identifying the species of leptospires as indicated by the clustering patterns up to species level (Figure 1). The tree generated gives an idea on the phylogenetic organization of the *Leptospira*. The *L. interrogans *seems to be like a clonal branch as the isolates are more closely related and emerge from *L. kirschneri *indicating that they have evolved from this species. The *L. interrogans *and the *L. kirschneri *emerge from *L. noguchii *branch indicating it as a monophyletic group [2]. Due to the greater sequence diversity observed in all the six genes except *rrs*2, the dendrogram generated could differentiate effectively the *L. interrogans*, *L. kirschneri*, *L. noguchii*, *L. santarosai *and *L. borgpetersenii*.

## Conclusion

With this new technique of MLST, we believe the issues related to ever-increasing serotype diversity would be effectively addressed *via *high throughput genome profiling. This will help establish population genetic structure of this pathogen with diverse host range and under different ecological conditions and will provide a scope for genotype-phenotype correlation to be established. Analyses based on the allelic profiles generated by our method may be successfully used to gain insights into the evolution and phylogeographic affinities of leptospires as it has been done for many other organisms. Large-scale, global genotyping, therefore, largely constitutes the essential mandate of studying leptospirosis in different hosts at the population level. Such approaches always generate extremely valuable information that can be translated into a wealth of databases to search for strain specific markers for epidemiology or to construct evolutionary history of the strains for a particular epidemiological catchment area. This task becomes greatly simplified if the genotypic data are categorized, stacked, archived and made electronically portable to facilitate easy access, extensive comparisons, remote access and retrieval in sets.

## Competing interests

The author(s) declare that they have no competing interests.

## Authors' contributions

NA and SMD carried out all the experiments related to primer designing, DNA sequencing and phylogenetic analyses and wrote the manuscript. NA and RAH designed the study and edited the manuscript. MDLAV, RSM, PV and WAE performed isolations of *Leptospira*. WAE and RAH performed serological and (other) molecular characterizations of the isolates, extracted DNA from isolates and reference strains and provided geographic and epidemiological data.

## References

[B1] Levett PN (2001). Leptospirosis. Clin Microbiol Rev.

[B2] Haake DA, Suchard MA, Kelley MM, Dundoo M, Alt DP, Zuerner RL (2004). Molecular Evolution and Mosaicism of Leptospiral Outer Membrane Proteins Involves Horizontal DNA Transfer. J Bacteriol.

[B3] Kmety E, Dikken H (1993). Classification of the species *Leptospira interrogans *and history of its serovars.

[B4] Brenner DJ, Kaufmann AF, Sulzer KR, Steigerwalt AG, Rogers FC, Weyant RS (1999). Further determination of DNA relatedness between serogroups and serovars in the family Leptospiraceae with a proposal for *Leptospira alexanderi *sp. nov. and four new *Leptospira *genomospecies. Int J Syst Bacteriol.

[B5] Ramadass P, Jarvis BDW, Corner RJ, Penny D, Marshall RB (1992). Genetic characterization of pathogenic *Leptospira *species by DNA hybridization. Int J Syst Bacteriol.

[B6] Yasuda BH, Steigerwalt AG, Sulzer LR, Kauhnann AF, Rogers F, Brenner DJ (1987). Deoxyribonucleic acid relatedness between serogroups and serovars in the family Leptospiraceae with proposals for seven new *Leptospira *species. Int J Syst Bacteriol.

[B7] Levett PN, Morey RE, Galloway RL, Steigerwalt AG (2006). *Leptospira broomii *sp. nov., isolated from humans with Leptospirosis. Int J Syst Evol Microbiol.

[B8] Savio ML, Rossi C, Fusi P, Tagliabue S, Pacciarini ML (1994). Detection and identification of *Leptospira interrogans *serovars by PCR coupled with restriction endonucleas eanalysis of amplified DNA. J Clin Microbiol.

[B9] Brown PD, Levett PN (1997). Differentiation of *Leptospira *species and serovars by PCR-restriction Endonuclease analysis, arbitrarily primed PCR and low-stringency PCR. J Med Microbiol.

[B10] Herrmann JL, Baril C, Belienger E, Perolat P, Baranton G, Girons IS (1991). Genome conservation in isolates of *Leptospira interrogans*. J Bacteriol.

[B11] Herrmann JL, Bellenger E, Perolat P, Baranton G, Girons IS (1992). Pulsed-field gel electrophoresis of NotI digests of leptospiral DNA: a new rapid method of serovars identification. J Clin Microbiol.

[B12] Zuerner RL, Herrmann JL, Girons IS (1993). Comparison of geneticmaps for two *Leptospira interrogans *serovars provides evidence for two chromosomes and intra species heterogeneity. J Bacteriol.

[B13] Perolat P, Merien F, Ellis WA, Baranton G (1994). Characterization of *Leptospira *isolates from serovars hardjo by Ribotyping, arbitrarily primed PCR, and mapped restriction site polymorphisms. J Clin Microbiol.

[B14] Majed Z, Bellenger E, Postic D, Pourcel C, Baranton G, Picardeau M (2005). Identification of variable-number tandem-repeat loci in *Leptospira interrogans *sensu stricto. J Clin Microbiol.

[B15] Vijayachari P, Ahmed N, Sugunan AP, Ghousunnisa S, Rao KR, Hasnain SE, Sehgal SC (2004). Use of Fluorescent Amplified Fragment Length Polymorphism for Molecular Epidemiology of Leptospirosis In India. J Clin Microbiol.

[B16] Enright MC, Spratt BG (1999). Multilocus sequence typing. Trends Microbiol.

[B17] Maiden MCJ, Bygraves JA, Spratt BG (1998). Multilocus sequence typing: a portable approach to the identification of clones within populations of pathogenic microorganisms. Proc Natl Acad Sci U S A.

[B18] Enright MC, Spratt BG (1998). A multilocus sequence typing scheme for *Streptococcus pneumoniae *: identification of clones associated with serious invasive disease. Microbiology.

[B19] Kotetishvili M, Kreger A, Wauters G, Morris JG, Sulakvelidze A, Stine OC (2005). Multilocus Sequence Typing for Studying Genetic Relationships among Yersinia Species. J Clin Microbiol.

[B20] Dingle KE, Colles FM, Wareing DRA, Ure R, Fox AJ, Bolton FE, Bootsma HJ, Willems RJL, Urwin R, Maiden MCJ (2001). Multilocus sequence typing system for *Campylobacter jejuni*. J Clin Microbiol.

[B21] Devi SM, Ahmed I, Khan AA, Rahman SA, Alvi A, Sechi LA, Ahmed N (2006). Genomes of *Helicobacter pylori *from native Peruvians suggest admixture of ancestral and modern lineages and reveal a western type cag-pathogenicity island. BMC Genomics.

[B22] Ko AI, Reis MG, Dourado CMR, Johnson WD, Riley LW (1999). Urban epidemic of severe Leptospirosis in Brazil. Lancet.

[B23] Nascimento AL, Ko AI, Martins EA, Monteiro-Vitorello CB, Ho PL, Haake DA, Verjovski-Almeida S, Hartskeerl RA, Marques MV, Oliveira MC, Menck CF, Leite LC, Carrer H, Coutinho LL, Degrave WM, Dellagostin OA, El-Dorry H, Ferro ES, Ferro MI, Furlan LR, Gamberini M, Giglioti EA, Goes-Neto A, Goldman GH, Goldman MH, Harakava R, Jeronimo SM, Junqueira-de-Azevedo IL, Kimura ET, Kuramae EE, Lemos EG, Lemos MV, Marino CL, Nunes LR, de Oliveira RC, Pereira GG, Reis MS, Schriefer A, Siqueira WJ, Sommer P, Tsai SM, Simpson AJ, Ferro JA, Camargo LE, Kitajima JP, Setubal JC, Van Sluys MA (2004). Comparative genomics of two *Leptospira interrogans *pathogenesis. J Bacteriol.

[B24] http://frodo.wi.mit.edu/.

[B25] Jeanmougin F, Thompson JD, Gouy M, Higgins DG, Gibson TJ (1998). Multiple sequence alignment with Clustal X. Trends Biochem Sci.

[B26] Jolley KA, Feil EJ, Chan MS, Maiden MC (2001). Sequence type analysis and recombinational tests (START). Bioinformatics.

[B27] Kumar S, Tamura K, Nei M (2004). MEGA3: Integrated software for Molecular Evolutionary Genetics Analysis and Sequence Alignment. Brief Bioinfor.

[B28] Slack AT, Dohnt MF, Symonds ML, Smythe LD (2005). Development of a Multiple-Locus Variable number of tandem repeat Analysis (MLVA) for *Leptospira interrogans *and its application to *Leptospira interrogans *serovars Australis isolates from Far North Queensland, Australia. Annals Clin Microbiol and Antimicrobiol.

[B29] Maiden MCJ (2000). High-throughput sequencing in the population analysis of bacterial pathogens of humans. Int J Med Microbiol.

